# Transcriptomic Analysis of the Effect of GAT-2 Deficiency on Differentiation of Mice Naïve T Cells Into Th1 Cells *In Vitro*

**DOI:** 10.3389/fimmu.2021.667136

**Published:** 2021-06-02

**Authors:** Xueyan Ding, Yajie Chang, Siquan Wang, Dong Yan, Jiakui Yao, Guoqiang Zhu

**Affiliations:** ^1^ College of Veterinary Medicine, Yangzhou University, Yangzhou, China; ^2^ Jiangsu Co-Innovation Center for Prevention and Control of Important Animal Infectious Diseases and Zoonoses, Yangzhou University, Yangzhou, China; ^3^ Joint International Research Laboratory of Agriculture and Agri-Product Safety, The Ministry of Education of China, Yangzhou University, Yangzhou, China; ^4^ Clinical Medical College, Yangzhou University, Yangzhou, China

**Keywords:** GAT-2 deficiency, Th1 cell differentiation, transcriptomic analysis, signal transduction, metabolic processes, qRT-PCR

## Abstract

The neurotransmitter γ-aminobutyric acid (GABA) is known to affect the activation and function of immune cells. This study investigated the role of GABA transporter (GAT)-2 in the differentiation of type 1 helper T (Th1) cells. Naïve CD4^+^ T cells isolated from splenocytes of GAT-2 knockout (KO) and wild-type (WT) mice were cultured; Th1 cell differentiation was induced and transcriptome and bioinformatics analyses were carried out. We found that GAT-2 deficiency promoted the differentiation of naïve T cells into Th1 cells. RNA sequencing revealed 2984 differentially expressed genes including 1616 that were up-regulated and 1368 that were down-regulated in GAT-2 KO cells compared to WT cells, which were associated with 950 enriched Gene Ontology terms and 33 enriched Kyoto Encyclopedia of Genes and Genomes pathways. Notably, 4 signal transduction pathways (hypoxia-inducible factor [HIF]-1, Hippo, phospholipase D, and Janus kinase [JAK]/signal transducer and activator of transcription [STAT]) and one metabolic pathway (glycolysis/gluconeogenesis) were significantly enriched by GAT-2 deficiency, suggesting that these pathways mediate the effect of GABA on T cell differentiation. Our results provide evidence for the immunomodulatory function of GABA signaling in T cell-mediated immunity and can guide future studies on the etiology and management of autoimmune diseases.

## Introduction

Gamma-aminobutyric acid (GABA) is the main inhibitory neurotransmitter in the central nervous system (CNS) of mammals and insects (1–4) and is responsible for regulating homeostasis, hormone secretion, sleep, and aging (5–10). There is increasing evidence that GABA also has an immunomodulatory function (11, 12). For example, GABA participates in T cell-mediated immunity through GABA transporters (GATs) and GABA receptors (11–18). GATs including GAT-1, GAT-2, GAT-3, and betaine/GABA transporter (BGT)-1 transport GABA into cells and reduce extracellular GABA concentration, thus inhibiting GABA signaling (19, 20). The transporters are highly selective Na^+^/Cl^−^-dependent glycoproteins that are potential drug targets in various diseases related to dysregulation of GABAergic transmission (12, 13, 21–23). GAT-2 is widely expressed in liver, kidney, lung, testicles, retina, immune cells, and intestinal cells (13).

T helper (Th) cells are key effectors of the adaptive immune response that differentiate into multiple subtypes including type 1 Th (Th1), Th2, Th17, T follicular helper (Tfh), and regulatory T (Treg) cells in response to T cell receptor (TCR) activation by cytokines (24, 25). Different Th cell lineages perform specific biological functions; for example, Th1 cells induce cellular immune responses to intracellular pathogens; Th2 cells participate in the clearance of extracellular pathogens; Th17 cells are involved in autoimmune diseases; and Treg cells inhibit hyperactivated T lymphocytes to maintain immune homeostasis (26–33). Tfh cells are located in lymphoid follicles of lymphatic organs where they secrete interleukin (IL)-21 and induce B cell activation and differentiation (34). Th cell subtypes cooperate to maintain homeostasis and their dysregulation can lead to the development of inflammation and autoimmune diseases (35, 36).

Our previous studies demonstrated that GAT-2 deficiency enhanced Th17 cell differentiation and responses in a mouse model through activation of GABA/mammalian target of rapamycin (mTOR) signaling (37, 38). However, the effect of GAT-2 deficiency on Th1 cell differentiation is unknown. In the present study we addressed this point through GAT-2 loss-of-function experiments using an *in vitro* model of mouse Th1 cell differentiation. We carried out gene expression profiling of differentiated Th1 cells from GAT-2 knockout (KO) and wild-type (WT) mice by RNA sequencing (RNA-seq)—a widely used high-throughput technique for transcriptome analysis that provides important information on gene expression and activation of signaling pathways (39)—and analyzed the functions of differentially expressed genes (DEGs).

## Materials and Methods

### Animals and Ethics Statement

Eight-week-old WT C57BL/6J mice (weighing 20 ± 2 g) were obtained from Yangzhou University (Yangzhou, China). GAT-2 KO mice (on a C57BL/6J genetic background) were a gift from Professor Wenkai Ren (South China Agricultural University, Guangzhou, China). The mice were allowed to acclimatize to the laboratory environment for 1 week before experiments, during which time they had free access to food and water. The mice were maintained in an environment with a temperature of 22°C–26°C, relative humidity of 40%–60%, on a 12:12-h light/dark cycle. The experiments complied with institutional animal care guidelines and were approved by the University of Yangzhou Animal Care Committee (no. YZUDWSY2017-0029) (40).

### Th1 Cell Preparation and Collection

WT and KO mice were euthanized with sodium pentobarbital (50 mg/kg body weight). The spleen was removed aseptically and passed through a 200-mesh sieve to dissociate the cells, which were centrifuged at 300×*g* for 7 min. The supernatant was discarded and red blood cell lysis buffer (cat. no. 555899; BD Biosciences; Franklin Lakes, NJ, USA) was added, followed by incubation for 4 min at room temperature. The cells were washed twice with sterile phosphate-buffered saline and centrifuged at 300×*g* for 5 min to obtain a single-cell suspension. Naïve cluster of differentiation (CD)4^+^CD62L^+^ T cells were isolated using a commercial kit (>95% purity, cat. no. 130-106-643; Miltenyi Biotec, Bergisch Gladbach, Germany) and stimulated with the following cytokines and antibodies to induce Th1 cell differentiation: anti-CD3ϵ (5 μg/ml, cat. no. 100313), anti-CD28 (2 μg/ml, cat. no. 102111), and anti–IL-4 (10 μg/ml, cat. no. 504122) antibodies (all from Biolegend, San Diego, CA, USA); and IL-2 (20 ng/ml, cat. no. 212-12-20) and IL-12 (20 ng/ml, cat. no. 210-12-10) (both from Peprotech, Rocky Hill, NJ, USA) at 37°C and 5% CO_2_. After 2 days, the anti-CD3ϵ and anti-CD28 antibodies were removed and the cells were cultured for another 3 days. At the end of the experiment, the cells were collected and immediately frozen in liquid nitrogen and stored at −80°C until use (38). The proportion of Th1 cells (CD4^+^ interferon [IFN]-γ^+^) was analyzed by flow cytometry using the following antibodies: fluorescein isothiocyanate-conjugated anti-mouse CD4 (cat. no. 100406), allophycocyanin (APC)-conjugated anti-mouse IFN-γ (cat. no. 505810), and APC-conjugated rat IgG1, κ isotype control (cat. no. 400412) (all from Biolegend).

### Library Generation and RNA-Seq

Total RNA was extracted from cells by adding 1 ml TRIzol reagent (Invitrogen Life Technologies, Carlsbad, CA, USA) and homogenizing the cells on ice. The samples were transferred to room temperature for 5 min before adding 200 μl chloroform for 10 min, followed by centrifugation at 12,000×*g* for 15 min. The supernatant was combined with 500 μl isopropanol and centrifuged at 12,000×*g* for 15 min. The RNA was washed with 75% ethanol, centrifuged at 7500×*g* for 15 min, and dissolved in 30 μl diethyl pyrocarbonate-treated water. RNA concentration was measured using the Qubit RNA Assay Kit on a Qubit 2.0 fluorometer (Invitrogen Life Technologies), and RNA integrity was evaluated with an RNA 6000 Nano Assay Kit on a Bioanalyzer 2100 system (Agilent Technologies, Santa Clara, CA, USA) (41).

mRNA was purified from total RNA samples using oligo (dT)-coupled magnetic beads and sheared into short fragments about 150–200 bp in length that were used as templates for cDNA synthesis. After end repair, A-tails were added to the cDNAs and sequencing adapters were attached. AMPure XP beads were used to screen cDNAs about 200 bp in length for PCR amplification and to purify the PCR products, yielding the final cDNA library. Clustering of the index-coded samples was performed on a cBot Cluster Generation System using the TruSeq PE Cluster Kit v3-cBot-HS (Illumina, San Diego, CA, USA). The constructed libraries were tested with a 2100 BioAnalyzer (Agilent Technologies) and ABI StepOnePlus Real-time PCR System (Applied Biosystems, Foster City, CA, USA) and sequenced with the Illumina Novaseq 6000 platform (42, 43).

### Quality Control and Reads Mapping

Raw image data files obtained with the Novaseq 6000 platform were transformed into sequenced reads by base calling using CASAVA software, yielding raw reads; those containing adapters or poly-N content >5% and those of low quality were removed. The high-quality data (clean reads) were saved in FASTQ format and Q20, Q30, and GC content were calculated. We downloaded the reference genome and gene model annotation files from the genome website (44). HISAT2 v2.0.5 (http://daehwankimlab.github.io/hisat2/) was used to construct the reference genome index and compare the paired-end clean reads with the reference genome.

### Identification and Functional Annotation of DEGs

The featureCounts program was used to obtain the read count of genes, and reads per kilobases per million mapped reads was used to qualify gene expression to eliminate the effects of gene length and inter-sample differences (45, 46). Differential expression analysis based on a negative binomial distribution was performed using DESeq2 R v1.16.1 software (47). The Benjamini–Hochberg method was used to adjust the P value to control the error detection rate. The adjusted P value was determined with DESeq2 and genes with P<0.05 were identified as DEGs based on |log_2_(fold change)|>0.0 and P<0.05 (48, 49).

After identifying DEGs, we used Metascape (http://metascape.org) for Gene Ontology (GO) and Kyoto Encyclopedia of Genes and Genomes (KEGG) enrichment analyses. The GO and KEGG pathway enrichment analyses were performed using the clusterProfiler package of R software with correction for gene length bias; GO terms with a corrected P value <0.05 were considered as significantly enriched (50, 51).

### Validating DEG Expression by Quantitative Real-Time (qRT)-PCR

In order to validate the transcriptome sequencing results, we selected 16 DEGs for verification by qRT-PCR as previously described (38, 41). Specific primers (**Table 1**) were designed according to reference sequences in the NCBI database with Primer-BLAST and were synthesized by Tsingke Biotech Co (Beijing, China). Total RNA was extracted using TRIzol reagent and reverse-transcribed into cDNA (KR118-02; Tiangen Biotech, Beijing, China) according to the manufacturer’s protocol. qRT-PCR was carried out on a 7500 Real Time System (Applied Biosystems). The 20-μl reaction contained 10 μl AceQ qPCR SYBR Green Master Mix (Low ROX Premixed) (cat. no. Q131-02; Vazyme, Nanjing, China), 2 μl cDNA template, 0.4 μl forward and reverse primers, and 7.2 μl nuclease-free deionized water. The 2-step method was used for amplification with predenaturation at 95°C for 5 min, followed by 40 cycles of 95°C for 10 s and 60°C for 30 s; and final elongation at 95°C for 15 s, 60°C for 1 min, and 95°C for 15 s. The mRNA expression levels of target genes were calculated with the 2^−ΔΔCt^ method using β-actin as internal reference. All samples were tested in triplicate.

**Table 1:** Primers used for qRT-PCR analysis.

Gene names	Accession numbers	Primers (5′-3′)	Product lengths (bp)
*β-actin*	NM_007393.3 (44)	Forward: GTCCACCTTCCAGCAGATGT Reverse: GAAAGGGTGTAAAACGCAGC	117
*Ifng*	NM_008337.4 (44)	Forward: GCTTTGCAGCTCTTCCTCA Reverse: CTTTTGCCAGTTCCTCCAG	153
*Areg*	NM_009704.4	Forward: TCCTCGCAGCTATTGGCATC Reverse: GTCATTTCCGGTGTGGCTTG	189
*Tead3*	NM_001204156.1	Forward: CTCCACGCAGGCCTTAGC Reverse: CAGAACTGTAGGGGACAGGC	149
*Wnt8a*	NM_009290.3	Forward: GGGAACGGTGGAATTGTCCT Reverse: CAGCCGCAGTTTTCCAAGTC	163
*Ppp2r2c*	NM_001360003.1	Forward: CACGGACACGCGGAAAATTA Reverse: GTCAGCTTCGGAGGGCATT	141
*Cxcr2*	NM_009909.3	Forward: ACTCTGCTCACAAACAGCGTC Reverse: TGAGTGGCATGGGACAGCAT	175
*Fcer1a*	NM_010184.2	Forward: TGCTGTTCATGTCTCTTGATGTC Reverse: AGGTGATTGTTCCCATAGCAGG	127
*Il13*	NM_008355.3	Forward: ATGGCCTCTGTAACCGCAAG Reverse: CCTCATTAGAAGGGGCCGTG	133
*Il5*	NM_010558.1	Forward: GACGAGGCAGTTCCTGGATT Reverse: TTGCACAGTTTTGTGGGGTT	155
*Il21*	NM_001291041.1	Forward: GACTCATAGGCTCTCGTTCCC Reverse: GTACTCCTGCATTCGTGAGC	144
*Il12a*	NM_001159424.2	Forward: CTCCCTTGGATCTGAGCTGG Reverse: GATTGACACATGCTGGACCG	160
*Camk2b*	XM_006514479.4	Forward: TGACCATCAACCCTGCCAAG Reverse: GGCTGCTGAGAAATTCCGTG	194
*Nos2*	NM_010927.4	Forward: GAAACTTCTCAGCCACCTTGG Reverse: GAAGAGAAACTTCCAGGGGCA	181
*Wnt10b*	NM_011718.2	Forward: GAACCACCCGTGAGTTAGGT Reverse: AGGGAGGGTAAAAGGGAGGG	102
*Amh*	NM_007445.3	Forward: CTCGGGCCTCATCTTAACCC Reverse: CGTGAAACAGCGGGAATCAG	103
*Grm6*	NM_173372.2	Forward: TCTCTGCCCTCACCCTCATC Reverse: CTGGACTGAGCCAACCTCTG	112

### Statistical Analysis

Data are presented as mean ± standard deviation and were analyzed using Prism v6.0 software (GraphPad, La Jolla, CA, USA). The unpaired *t* test was used for data that conformed to a Gaussian distribution and had equal variance, and the unpaired *t* test with Welch’s correction was used for data that followed a Gaussian distribution but showed unequal variance. Data that were non-normally distributed were analyzed with the nonparametric Mann–Whitney *U* test. P<0.05 was considered significant for all tests.

## Results

### GAT-2 Deficiency Promoted Th1 Cell Differentiation

**Figure 1:**
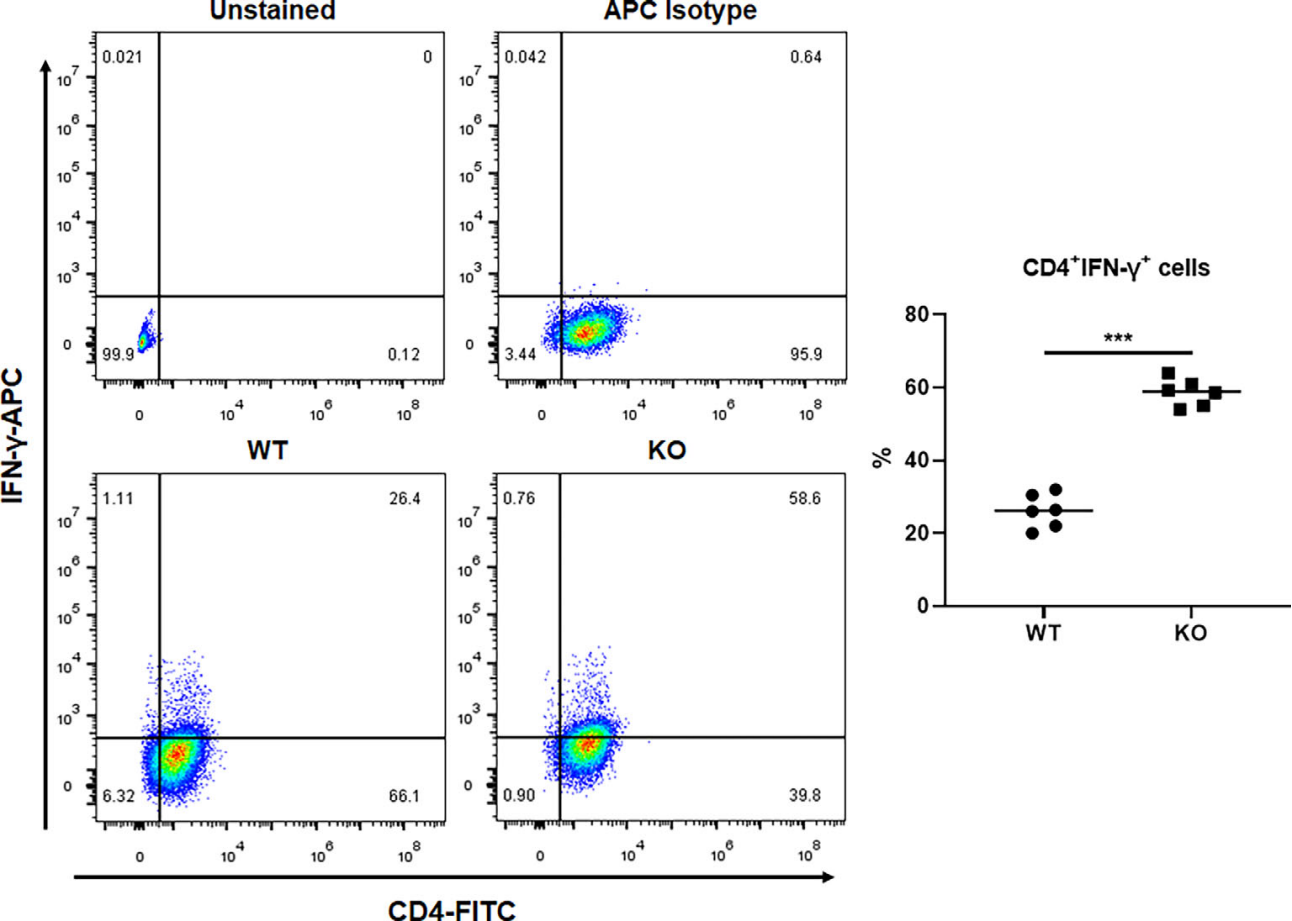
Flow cytometry analysis of percentages of Th1 cells differentiated from WT and KO naïve CD4^+^ T cells. Data were analyzed by unpaired *t*-test and shown as the means ± SD (*n*=6). ***P <0.001.

We investigated the effect of GAT-2 deficiency on Th1 cell differentiation using naïve CD4^+^ T cells from WT and GAT-2 KO mice cultured in the presence of specific cytokines and antibodies. A larger percentage of Th1 cells were differentiated from CD4^+^ T cells from KO mice as compared to WT mice (**Figure 1**), indicating that GAT-2 deficiency enhanced Th1 cell differentiation.

### Sequencing and Transcriptome Assembly

After constructing qualified libraries, RNA-seq was performed using the Illumina platform. WT and GAT-2 KO RNA libraries obtained by RNA-seq contained 51,920,523 and 52,155,649 short reads, respectively (**Table 2**). After filtering out low-quality data, 50,143,129 and 50,335,423 clean reads, respectively, were retained for a total of 7.52 Gb and 7.55 Gb clean bases, respectively. An analysis of the GC content of the sequences showed that the libraries were of excellent quality.

**Table 2:** Principal features of tags in two libraries and data of sequencing reads mapping to the reference genome.

Sample names	WT	KO
Raw reads	51 920 523	52 155 649
Clean reads	50 143 129	50 335 423
Clean bases	7.52G	7.55G
Q20 (%)	97.98	97.75
Q30 (%)	94.30	93.78
GC content (%)	49.09	48.95
Total mapped	48 333 131 (96.39%)	48 259 228 (95.90%)
Uniquely mapped	46 033 706 (91.81%)	45 863 013 (91.14%)
Reads map to ‘+’	23 006 053 (45.88%)	22 919 570 (45.55%)
Reads map to ‘−’	23 027 654 (45.93%)	22 943 444 (45.59%)
Non-splice reads	29 153 218 (58.14%)	29 101 863 (57.85%)
Splice reads	67 521 951 (33.67%)	15 261 151 (33.30%)

‘+’ refers to sense strands; ‘−’ refers to anti-sense strands.

‘Non-splice reads’ refers to reads for the entire sequence is mapped to one exon; ‘Splice reads’, also called ‘junction reads’, refers to reads mapped to the border of exon.

### Mapping Reads to the Transcriptome

As sequenced fragments were randomly interrupted, clean reads were mapped to the reference genome to identify the corresponding genes. Most of the clean reads (96.39% and 95.90% in WT and KO libraries, respectively) matched the reference genome, and a large portion could be uniquely mapped to the *Mus musculus* genome including 46,033,706 (91.81%) and 45,863,013 (91.14%) reads in the WT and KO libraries, respectively (**Table 2**). Moreover, in each sample nearly 58% of clean reads were unspliced. These data demonstrated that the genome assembly of our samples was relatively complete and was consistent with the reference genome, and that there was no sample contamination during the experiments.

### Differential Expression Analysis

**Figure 2:**
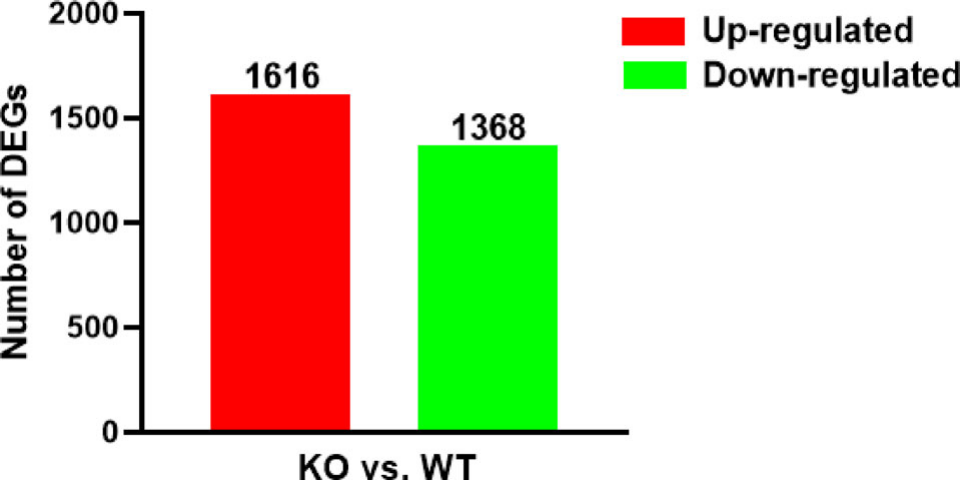
Numbers of up-/down-regulated DEGs in two contrasts (KO *vs* WT). The red bars represented genes that were up-regulated in KO compared to WT, while the green bars represented genes that were down-regulated.

After gene expression quantification was completed, the data needed to be statistically analyzed to screen the genes with significantly different expression levels between WT and KO groups. We screened genes with significantly different expression levels between the 2 groups based on the criteria |log_2_(fold change)|>0.0 and P<0.05. There were 2984 DEGs between the WT and KO groups including 1616 that were up-regulated and 1368 that were down-regulated in the KO group compared to the WT group (**Figure 2**). The full list of DEGs was shown in **Supplementary Table 1**.

### Functional Enrichment Analysis of DEGs

**Figure 3:**
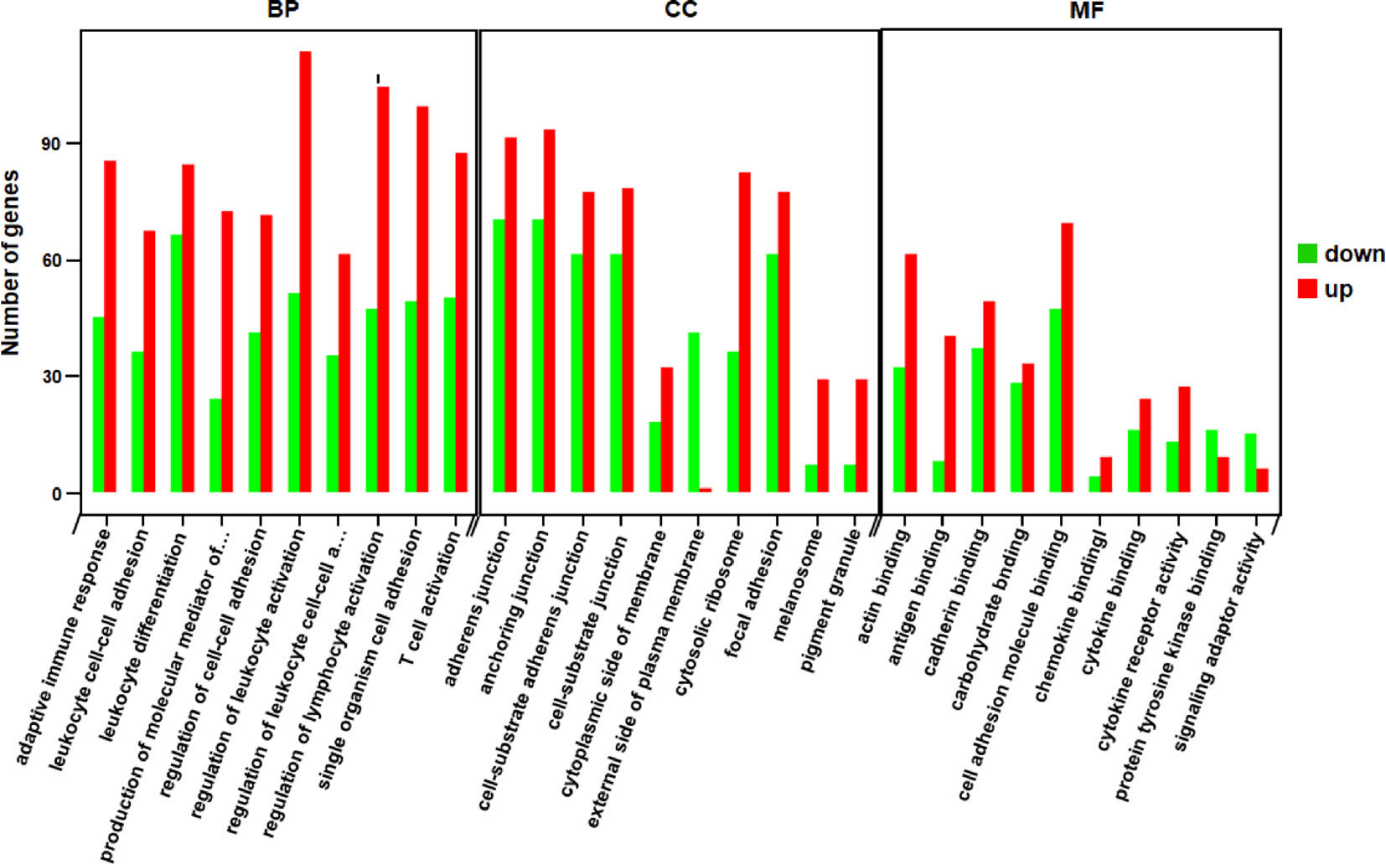
Functional GO categories of DEGs (KO *vs* WT). The red bars represented the number of genes that were up-regulated in KO compared to WT, while the green bars represented the number of genes that were down-regulated. BP, biological process; CC, cellular component; MF, molecular function.

**Figure 4:**
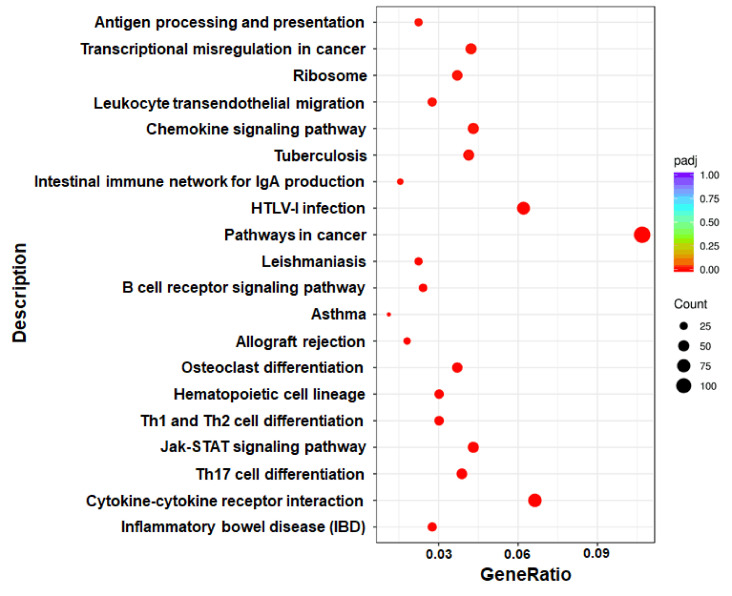
Scatter plot of DEGs enriched in KEGG pathways (KO *vs* WT). The GeneRatio represents the ratio of the number of DEGs to the number of all the unigenes in the pathway; the *padj* value represents the corrected P-value.

Through the enrichment analysis of differential gene sets, the biological functions and pathways deriving from DEGs under different conditions can be identified. Therefore, we carried out GO functional enrichment analysis and KEGG pathway enrichment analysis *via* Metascape (http://metascape.org) to identify the associated biological functions or pathways. In the GO analysis, gene functions were divided into 3 categories—namely, biological process (BP), cellular component (CC), and molecular function (MF). The threshold value for significant enrichment of a GO term was P<0.05. The top 30 most highly enriched GO terms in KO *vs* WT groups included adaptive immune response, T cell activation, cytokine receptor activity and other terms (**Figure 3**); the full list was shown in **Supplementary Table 2**. We also identified 33 significantly enriched KEGG pathways in the KO group *vs* the WT group. The top 20 are shown in **Figure 4**; these included Th1 and Th2 cell differentiation, Janus kinase (JAK)/STAT signaling, cytokine–cytokine receptor interaction, etc. Detailed information on the significantly enriched KEGG pathways was shown in **Supplementary Table 3**.

### Signal Transduction Pathways Related to Th1 Cell Differentiation

**Figure 5:**
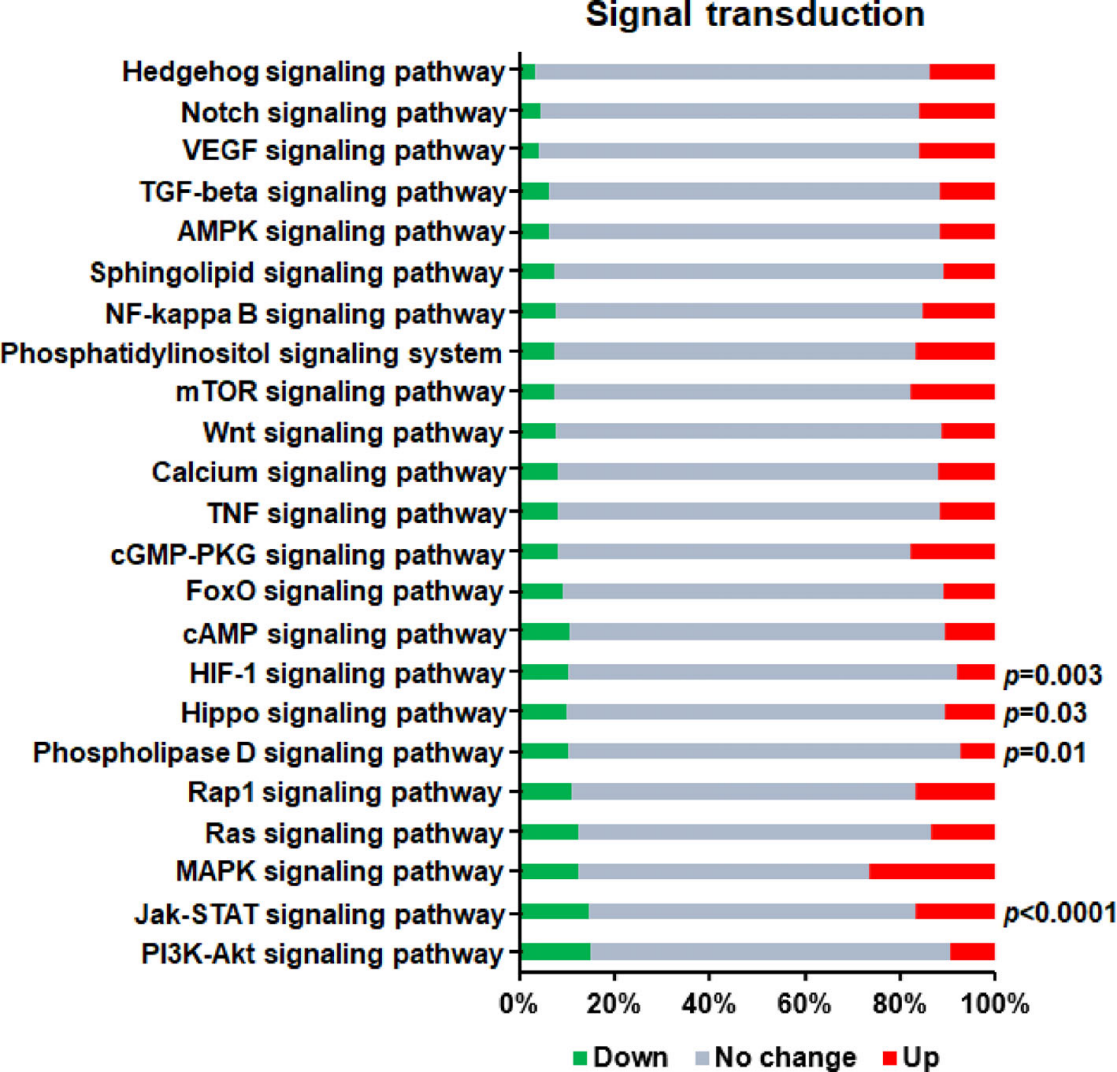
KEGG enrichment analysis of DEGs in signal transduction pathways related to Th1 cell differentiation. Red bars represent up-regulated genes, green bars represent down-regulated genes, and gray bars represent unchanged genes.

Signal transduction usually refers to the process that cells receive external signals through cell surface receptors, and then transfer the extracellular signals into intracellular signals through cascading transmission mechanism, and finally trigger a series of biochemical reactions inside the cells. It is now known that there are a variety of signal transduction pathways in cells to form a complex network, which can change the cellular metabolic process, thus affecting the growth and death rate of cells. Therefore, elucidating the mechanism of cell signal transduction is helpful to understand various aspects of cell proliferation, differentiation, metabolism and death. In this study, in order to find out the signal transduction pathways that regulate the differentiation process of Th1 cells, we investigated the signal transduction pathways that mediate the effect of GAT-2 deficiency on Th1 cell differentiations identified by KEGG pathway analysis (**Figure 5**). We found that four signal transduction pathways, including “HIF-1 signaling pathway”, “Hippo signaling pathway”, “Phospholipase D signaling pathway”, and “Jak-STAT signaling pathway”, were significantly enriched (P<0.05), which suggested that the signal transduction system of Th1 cells was significantly altered after GAT-2 deficiency.

### Metabolic Processes Related to Th1 Cell Differentiation

**Figure 6:**
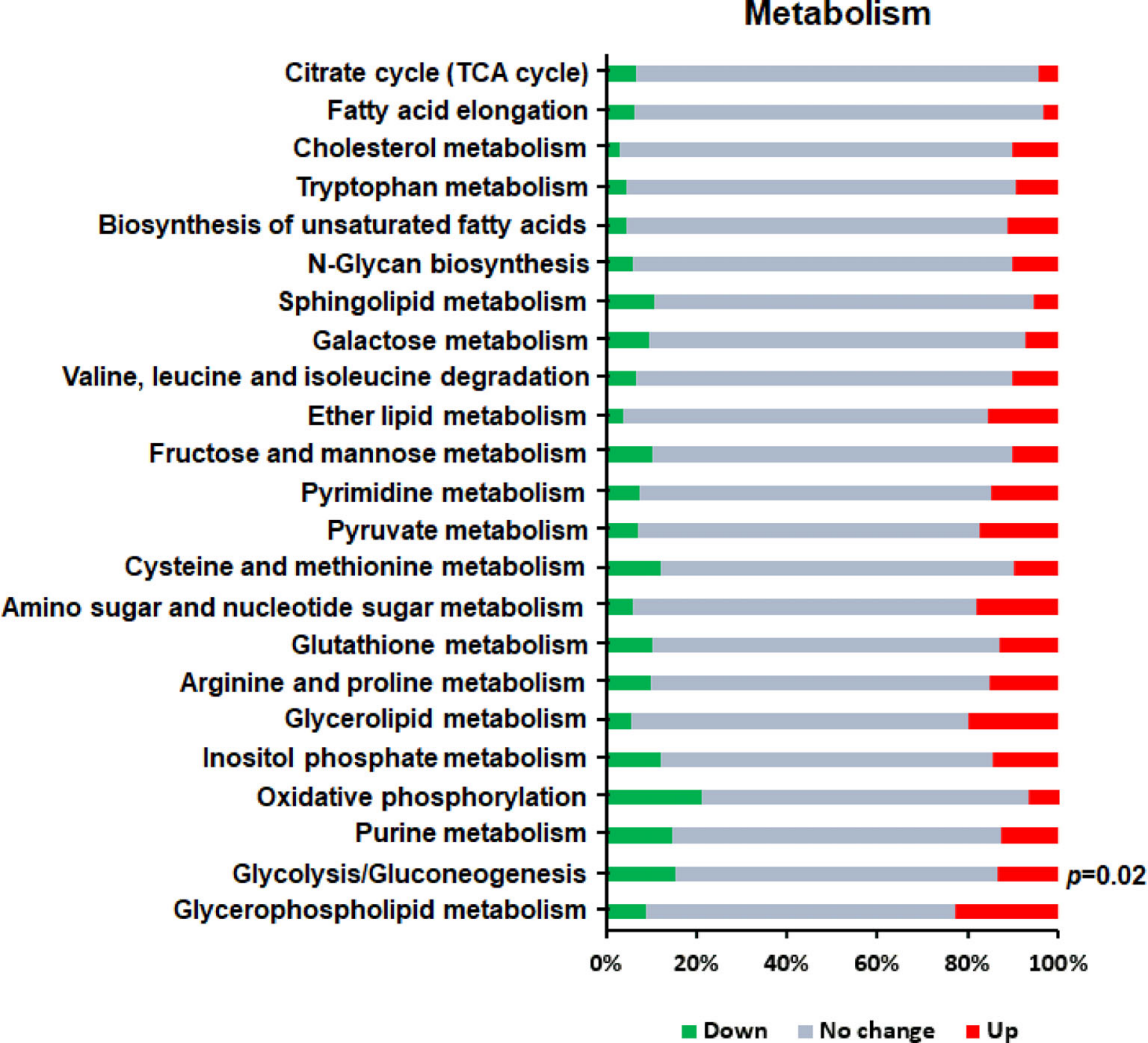
KEGG enrichment analysis of DEGs in metabolism related to Th1 cell differentiation. Red bars represent up-regulated genes, green bars represent down-regulated genes, and gray bars represent unchanged genes.

Cell metabolism is the general term for the ordered series of chemical reactions that take place in cells to maintain growth, proliferation and ability responding to the environment. Considering that after a gene is knocked out, changes in all aspects of the cell tend to occur simultaneously. Therefore, we also analyzed the intracellular metabolic processes related to Th1 cell differentiation and found only one significantly enriched KEGG pathway (P<0.05) (**Figure 6**)—namely, glycolysis/gluconeogenesis, which is an important aspect of carbohydrate metabolism and is involved in cellular growth, proliferation, and differentiation.

### Validation of DEGs Related to Signal Transduction and Metabolism by QRT-PCR

**Figure 7:**
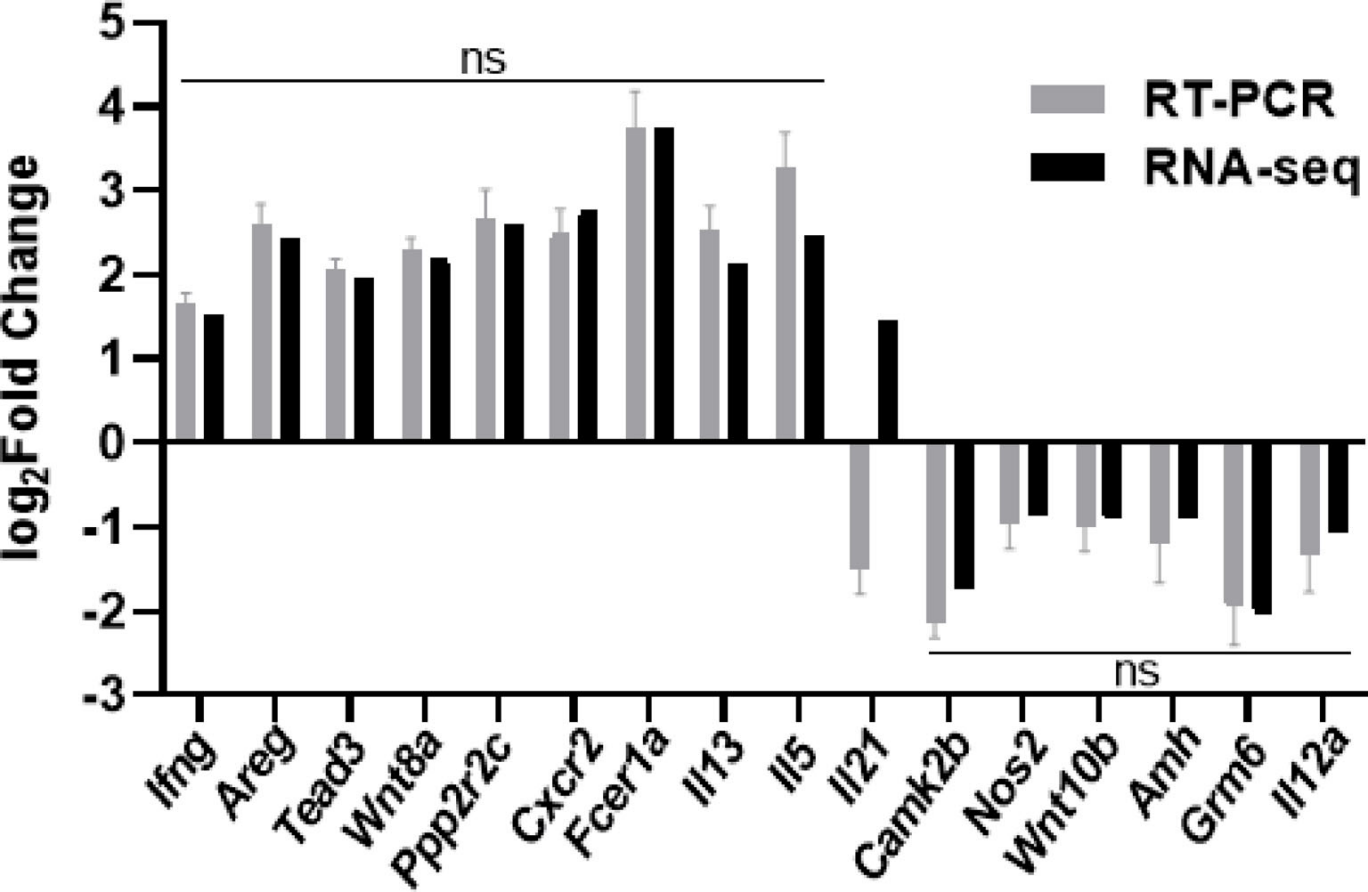
qRT-PCR validation of select differentially expressed genes by RNA-seq. Sixteen individual genes involved in signal transduction and metabolism were analyzed by qRT-PCR. The log_2_Fold Change determined from the relative Ct values of sixteen genes were compared to those detected by RNA-seq method. The black bar represents the expression levels analyzed by RNA-seq, and the grey bar represents the expression levels validated by qRT-PCR. Replicates (*n=*3) of each sample were run and all the Ct values were normalized to *β*-actin. Data were analyzed by Mann-Whitney test and shown as the means ± SD. ns, no significance.

To validate the reliability of RNA-seq analysis, we randomly selected 16 DEGs for validation by qRT-PCR (**Table 1**) and found that all of the genes showed consistent expression between the qRT-PCR and RNA-seq analyses, except for the gene encoding IL-21 (**Figure 7**). And there were strong correlations between qRT-PCR and RNA-seq results and the Pearson correlation coefficients (*R* ^2^) was 0.984, which confirmed the reliability and accuracy of the transcriptome data.

## Discussion

The GABAergic system, which includes GABA, GABA receptors, glutamate decarboxylase (GAD), vesicular inhibitory amino acid transporter (VIAAT), GABA transporters (GATs) and GABA transaminase (GABA-T), shows significant inhibitory functions in the CNS. Besides the CNS, GABA signaling plays an important role in the immune system (8, 11, 12, 52). For example, GAD and GABA receptors have been detected in macrophages and T cells (11), and GABA was shown to promote intestinal Th17 cell differentiation in the inflammation models of piglets and mice (37). Thus, this aroused great interest in exploring the role of the GABAergic system in activation and function of immune cells.

GATs terminate GABA signaling by mediating the translocation of GABA from the extracellular to the intracellular space (13). It has been proven that T cells express GAT-2 and GAT-2 deficiency promoted the Th17 cell response through the activation of GABA-mTOR signaling in the mouse infection model (37, 38). Here, in order to clarify the role of GAT-2 in T cell-mediated immunity, naïve CD4^+^ T cells were isolated from GAT-2 KO and WT mice and induced for Th1 cell differentiation, and then transcriptomic analysis was performed by RNA-seq. We found that GAT-2 deficiency enhanced the differentiation of naïve T cells into Th1 cells. Thus, to further explore the molecular mechanisms by which GAT-2 deficiency promoted Th1 cell differentiation, we next used the RNA-seq method to analyze the transcripts of differentiated Th1 cells and analyzed the data of transcriptomic sequencing.

In the present study, a mean of 50,239,276 clean reads were obtained per library (**Table 2**). This was sufficient to provide sequence coverage for transcriptional analysis, and the transcriptome sequencing data revealed that four signal transduction pathways (HIF-1 signaling pathway, Hippo signaling pathway, Phospholipase D signaling pathway, and Jak-STAT signaling pathway) were significantly enriched in GAT-2 KO cells compared to WT cells, suggesting that they are important for GABA signaling in T cells. Th cells undergo differentiation under hypoxic conditions in secondary lymphoid tissues; thus, their effector function is influenced by O_2_ concentrations (53). HIF-1 is a key transcription factor in the cellular response to hypoxia (54) and represses the transcription of STAT4 (a pro-Th1 factor), thereby inhibiting Th1 function *in vitro* (24). The Hippo signaling pathway was first discovered in *Drosophila*, which is evolutionally conserved and plays an important role in the regulation of cell proliferation and differentiation (55, 56). In multiple cell types of invertebrates and vertebrates, terminal differentiation is directed by the Hippo pathway. In mammalian tissues, macrophage stimulating (MST)1 and MST2—key kinases of the Hippo signaling pathway—control cell proliferation, differentiation, and apoptosis by inhibiting the transcriptional coactivator yes-associated protein (YAP). Phospholipase D activity is increased in activated T lymphocytes, which may result from disruption of the TCR/CD3 complex. Receptor-mediated phospholipase D activity was shown to be involved in human T lymphocyte activation (57). The JAK/STAT pathway, one of the most important signaling axes that regulate Th1 cell differentiation, is composed of transmembrane cytokine receptors, non-transmembrane proteins JAKs, and nucleoprotein STATs. The JAK/STAT signaling pathway is regulated by different cytokines and mediates a series of physiological and pathological processes such as cell proliferation, differentiation, migration and apoptosis (58). The differentiation of Th1 cells is regulated by a specific transcription factor T-bet, and the differentiation process is accomplished through JAK/STAT signaling pathway (59, 60). When naïve T cells differentiate into Th1 cells, IL-12 binds to its receptor on the surface of naïve T cells and activates STAT4 signaling *via* JAK to promote the differentiation of Th1 cells. At the same time, the expression of T-bet was also shown to be indirectly up-regulated to maintain the activation of STAT4 signaling, thus forming a positive feedback loop to ensure the continuous differentiation and proliferation of Th1 cells (61).

Metabolic reprogramming plays an important role in the activation, differentiation and function of T cells. In this study, we found that glycolysis/gluconeogenesis metabolic pathway was significantly enriched in GAT-2 KO cells compared to WT cells. Glycolysis is activated in rapidly proliferating tumor cells and immune cells including T cells and is associated with CD28 activation (62, 63). Upon T cell activation, cells shift from oxidative phosphorylation to glycolysis to meet biosynthetic and bioenergetic demands (64–66). The polarization of naïve CD4^+^ T cells depends on the enhancement of glycolysis, which also regulates T cell growth, differentiation, and function and stimulates the production of the pro-inflammatory cytokine IFN-γ and Th1 differentiation (67–69). In this study, multiple genes encoding components of the glycolysis/gluconeogenesis pathway (eg, phosphofructokinase, platelet [*pfkp*], aldolase C, fructose-bisphosphate [*aldoc*], enolase [*eno*]*1*, and *eno2*) were up-regulated in GAT-2 KO mice, which was consistent with our finding that GAT-2 deficiency enhanced Th1 differentiation. Increased glycolysis is a feature of activated T cells and promotes T cell differentiation *via* a epigenetic mechanism involving histone acetylation (70). Apparently, there is a need for more detailed data analyses and investigations on other changes that influence Th1 cell differentiation.

Interestingly, the current results inspired us to focus on signal transduction pathways and metabolic pathways for verification in our future work, so as to clarify the influence of GAT-2 deficiency on the signal transduction and metabolic activities of T cells. In addition, the combination of metabonomics, proteomics and other high-throughput sequencing technologies could be considered to comprehensively elucidate the more detailed regulatory network within T cells after GAT-2 deficiency.

## Conclusions

In summary, we found that GAT-2 deficiency promoted Th1 cell differentiation, which was associated with changes in multiple signal transduction pathways and metabolic processes. These findings provide insight into the role of GABA signaling in T cell-mediated immunity, which can guide future investigations on the etiology and management of autoimmune diseases.

## Data Availability Statement

The datasets presented in this study can be found in online repositories. The names of the repository/repositories and accession number(s) can be found below: NCBI SRA; PRJNA702918.

## Ethics Statement

The animal study was reviewed and approved by The Institutional Animal Care and Use Committee of Yangzhou University.

## Author Contributions

GZ and XD designed the experiments. XD performed the experiments and wrote the paper. YC, SW, and DY contributed to the experiments and data analysis. GZ and JY revised the manuscript. All authors contributed to the article and approved the submitted version.

## Funding

This project was supported by the Postgraduate Research & Practice Innovation Program of Jiangsu Province (grant no. KYCX19_2116); a project founded by the Priority Academic Program of Development Jiangsu High Education Institution; Programs from the Ministry of Science and Technology of the People’s Republic of China (grant no. 2016YFD0500905 and 2017YFD0500203); and International Collaboration Program from Science and Technology Agency of Jiangsu Province (2019).

## Conflict of Interest

The authors declare that the research was conducted in the absence of any commercial or financial relationships that could be construed as a potential conflict of interest.
